# Supplementary material for: Comprehensive Annotation and Functional Exploration of MicroRNAs in Lettuce
Source: Front Plant Sci. 2021 Dec 24;12:781836. doi: 10.3389/fpls.2021.781836 (PMC8739914; doi:10.3389/fpls.2021.781836)
Supplement: Supplementary file 2 [file Data_Sheet_8.ZIP › GSM2230750.plot/Lsa-miR160b_Lsat_1_v5_gn_2_100181.1_2076_TPlot.pdf]

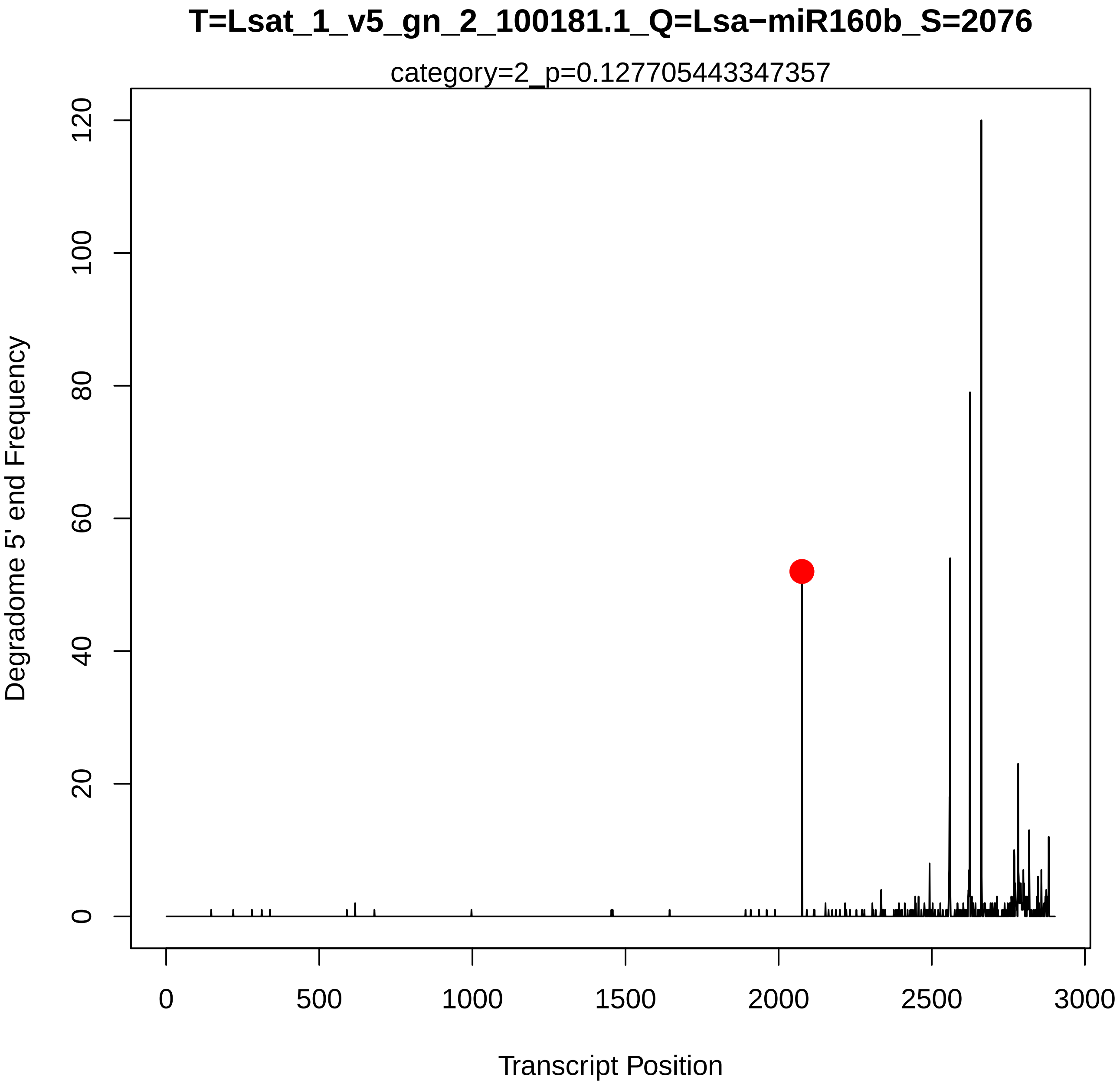

T=Lsat_1_v5_gn_2_100181.1_Q=Lsa−miR160b_S=2076
category=2_p=0.127705443347357
Degradome 5' end Frequency
0
20
40
60
80
100
120
0
500
1000
1500
2000
2500
3000
Transcript Position
